# Management of a Bronchopleural Fistula After Right-Sided Lobectomy Using a Delayed Random Flap Under Guidance of Indocyanine Green (ICG) Angiography: A Case Report

**DOI:** 10.7759/cureus.24536

**Published:** 2022-04-27

**Authors:** Robert J Dabek, Klara Schwarzova, Harrison McUmber, Daniel N Driscoll

**Affiliations:** 1 General Surgery, Ascension St. Agnes Hospital, Baltimore, USA; 2 Plastic, Reconstructive, and Laser Surgery, Shriners Hospitals for Children, Boston, USA; 3 Plastic Surgery, Tufts University School of Medicine, Boston, USA; 4 Division of Plastic and Reconstructive Surgery, Massachusetts General Hospital, Boston, USA

**Keywords:** icg angiography, indocyanine green (icg), random flap, bronchopleural fistula, fasciocutaneous flap, plastic surgery

## Abstract

Bronchopleural fistula (BPF) following lung resection and thoracic surgery is associated with high rates of morbidity and mortality. Various methods are available for the closure of BPF and thoracic dead space, including flap procedures and thoracoplasty. While delayed random flaps have been used for the treatment of BPF and closure of thoracic dead space, no previous reports have described the concurrent use of laser-assisted indocyanine green angiography (ICG-A). We report a case of successful BPF closure with a random delayed fasciocutaneous flap using laser-assisted ICG-A guidance for flap delay.

## Introduction

Bronchopleural fistula (BPF), abnormal communication between bronchi and pleura space, following lung resection is associated with high rates of morbidity and mortality. Various methods are available for the closure of BPF and thoracic dead space, including flap procedures and thoracoplasty [1-3]. While delayed random flaps have been used for the treatment of BPF and closure of thoracic dead space, no previous reports have described the concurrent use of laser-assisted indocyanine green angiography (ICG-A). We report a case of successful BPF closure with a random delayed fasciocutaneous flap using laser-assisted ICG-A guidance for flap delay.

## Case presentation

**Figure 1:**
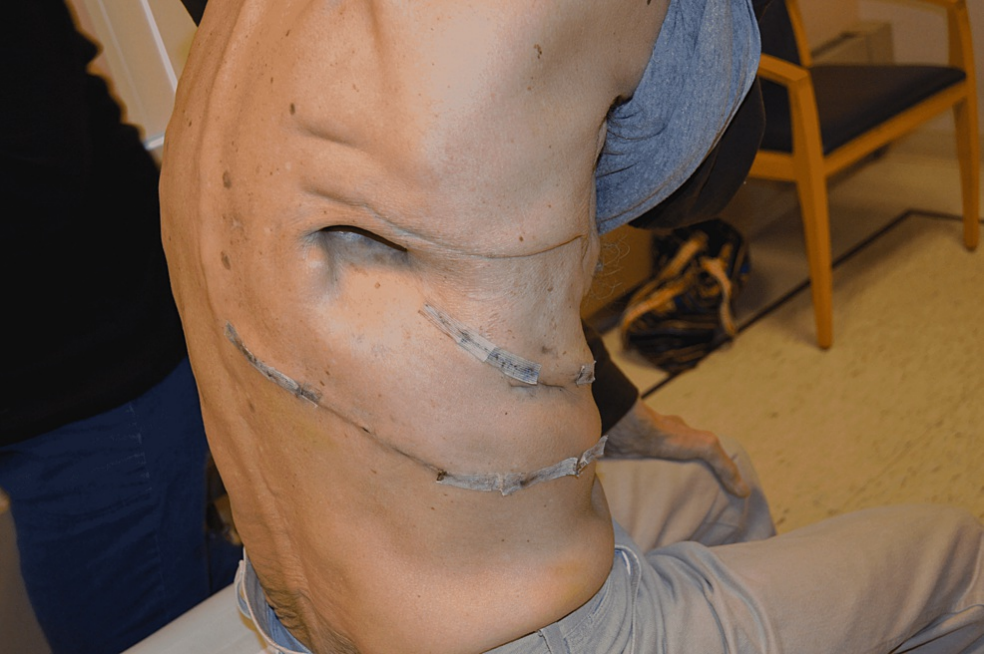
Persistent bronchopleural fistula after Eloesser procedure and multiple attempts to close.

A 72-year-old male presented to our clinic following a right lower lobe lobectomy for cancer treatment. He had a severe postoperative infection, empyema, and developed a BPF. He was transferred to a tertiary hospital for care of the BPF. He underwent conservative management and an Eloesser flap to open the space. They failed to close after several attempts (Figure 1). To increase the chances of successful closure of the fistula and the thoracic dead space, soft tissue was required to close the oversewn base. Although thoracoplasty was an option, it comes with a high risk of morbidity. Muscular flap closure using the latissimus muscle would be suboptimal because the latissimus was divided at the time of the thoracotomy. Microsurgical options existed as well (transverse rectus abdominis muscle (TRAM) flap, omental flap, serratus anterior flap, etc.), but incur significant donor site morbidity.

**Figure 2:**
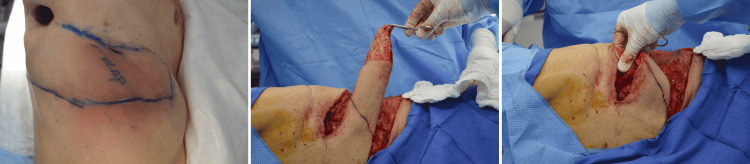
Marking of flap, elevation and deepithelialization, and rotation of flap.

**Figure 3:**
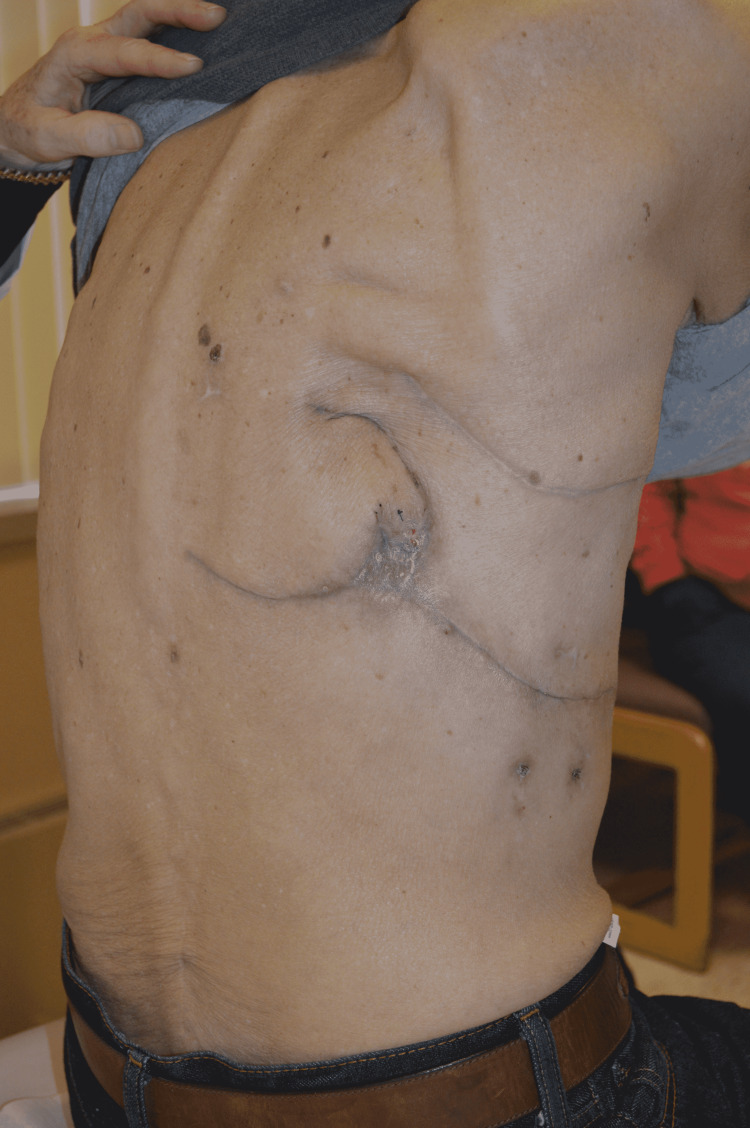
Approximately 5 weeks after the flap inset and closure of defect.

Opting for simplicity, we felt the best option was a posteriorly based fasciocutaneous flap rotated into position (Figure 2). As this is a random flap, having no named arterial axial blood supply, it required a delay. At the time of bronchoscopies and attempts at oversewing the fistula, we delayed the flap on two occasions and evaluated the blood supply using laser-assisted ICG-A. ICG dye 4 mL was administered intravenously, and cutaneous perfusion was observed for three minutes. At the time of the two delay procedures, sluggish perfusion was noted in the mid and distal portions of the flap, with limited or no perfusion at the distal tip (Video 1). Once there was evidence of good distal perfusion, and the flap was likely to survive on its skin pedicle, it was de-epithelialized and rotated into the defect. Two large drains were placed into the thoracic cavity, and the donor site was closed primarily. The ICG-A ensured that only viable flap tissue would be placed deeply against the BPF to give the best chance of closure and least chance of postoperative flap compromise and infection. The drains remained in place for three weeks and the BPF was closed. Subsequent patient follow-ups at 3 and 5-weeks showed adequate wound closure and no further complications or symptoms (Figure 3).

**Video 1:** ICG-enhanced perfusion of random skin flap. ICG: indocyanine green

## Discussion

Intrathoracic defects present several unique challenges. In particular, the problems associated with persistent dead space and BPF, which provide a continuous leak, make flap adherence and surgical intervention especially difficult and problematic. Although BPFs are rare and infrequent, they are a life-threatening complication in lung surgery, with high rates of morbidity and mortality [1,4,5].

BPFs are most commonly managed through bronchoscopy, appropriate drainage, irrigation, and surgical repair, which could include the use of a flap; delayed or non-delayed [2]. Surgical techniques under the guidance of plastic and reconstructive surgery have allowed for filling these defects with more distant flaps, rather than solely relying on locoregional flaps. In this case, a fasciocutaneous flap was used, as the traditionally used muscle flap was divided during the initial procedure. Fortunately, fasciocutaneous flaps have been shown to have long vascular pedicles that enable transposition and coverage of defects that cannot be reached by the conventional musculocutaneous flaps. Although traditional musculocutaneous flaps offer a more rapid and robust solution, they are often associated with more significant donor site morbidity than random locoregional fasciocutaneous flaps. The concept of a delayed procedure has been used by surgeons for roughly 4000 years [6]. The surgical intervention is performed such that tissues experience a reduction of perfusion, triggering angiogenesis. The goal is to improve blood flow through the subpapillary and cutaneous plexus.

Prior investigations have demonstrated the use of ICG imaging for vascular delay technique; [7] however, there are no clinical reports describing vascular assessment of a delayed flap using SPY (NOVADAQ Technologies, Inc., USA) and ICG in the management and treatment of BPF post-lobectomy. Recently, it has been reported that ICG was used successfully in determining the viability and perfusion of divided latissimus dorsi muscles used for chest wall reconstruction [3]. In addition, ICG has been used to evaluate pedicled intercostal flaps for BPF closure [8]. The introduction of ICG into reconstructive surgery has allowed surgeons to definitively determine the degree of flap perfusion, which is key to the clinical decision-making process in determining flap delay times and in ensuring the vitality of the flap. In this case, the concurrent use of ICG as well as the delayed flap technique prevented the recurrence of the opening of the fistula and helped avoid the development of complications from a persistent BPF. Additional case reports regarding the successful closure of complicated BPFs using this technique are needed to augment our findings.

## Conclusions

In conclusion, this case report describes for the first time the successful management of a post-lobectomy BPF using both a delayed flap technique and assessment under ICG guidance. This management technique should be further studied and standardized for use in other patient cases in hopes of minimizing morbidity and mortality from complex cases of BPFs.
